# Markers of Iron Metabolism and Outcomes in Patients with Heart Failure: A Systematic Review

**DOI:** 10.3390/ijms24065645

**Published:** 2023-03-15

**Authors:** Simrat Dhaliwal, Andreas P. Kalogeropoulos

**Affiliations:** Division of Cardiology and Department of Medicine, Renaissance School of Medicine, Stony Brook University, Stony Brook, NY 11794, USA; simrat.dhaliwal@stonybrookmedicine.edu

**Keywords:** iron metabolism, biomarkers, heart failure, outcomes

## Abstract

Iron deficiency (ID) in conjunction with heart failure (HF) poses a challenge for clinicians and is associated with worse HF outcomes. Treatment of ID with IV iron supplementation for patients with HF has demonstrated benefits in quality of life (QoL) and HF-related hospitalizations. The aim of this systematic review was to summarize the evidence linking iron metabolism biomarkers with outcomes in patients with HF to assist in the optimal use of these biomarkers for patient selection. A systematic review of observational studies in English from 2010 to 2022 was conducted using PubMed, with keywords of “Heart Failure” and respective iron metabolism biomarkers (“Ferritin”, “Hepcidin”, “TSAT”, “Serum Iron”, and “Soluble Transferrin Receptor”). Studies pertaining to HF patients, with available quantitative data on serum iron metabolism biomarkers, and report of specific outcomes (mortality, hospitalization rates, functional capacity, QoL, and cardiovascular events) were included, irrespective of left ventricular ejection fraction (LVEF) or other HF characteristics. Clinical trials of iron supplementation and anemia treatment were removed. This systematic review was conducive to formal assessment of risk of bias via Newcastle-Ottawa Scale. Results were synthesized based on their respective adverse outcomes and iron metabolism biomarker(s). Initial and updated searches identified 508 unique titles once duplicates were removed. The final analysis included 26 studies: 58% focused on reduced LVEF; age range was 53–79 years; males composed 41–100% of the reported population. Statistically significant associations of ID were observed with all-cause mortality, HF hospitalization rates, functional capacity, and QoL. Increased risk for cerebrovascular events and acute renal injury have also been reported, but these findings were not consistent. Varying definitions of ID were utilized among the studies; however, most studies employed the current European Society of Cardiology criteria: serum ferritin < 100 ng/mL or the combination of ferritin between 100–299 ng/mL and transferrin saturation (TSAT) < 20%. Despite several iron metabolism biomarkers demonstrating strong association with several outcomes, TSAT better predicted all-cause mortality, as well as long-term risk for HF hospitalizations. Low ferritin was associated with short-term risk for HF hospitalizations, worsening functional capacity, poor QoL, and development of acute renal injury in acute HF. Elevated soluble transferrin receptor (sTfR) levels were associated with worse functional capacity and QoL. Finally, low serum iron was significantly associated with increased risk for cardiovascular events. Considering the lack of consistency among the iron metabolism biomarkers for association with adverse outcomes, it is important to incorporate additional biomarker data, beyond ferritin and TSAT, when assessing for ID in HF patients. These inconsistent associations question how best to define ID to ensure proper treatment. Further research, potentially tailored to specific HF phenotypes, is required to optimize patient selection for iron supplementation therapy and appropriate targets for iron stores replenishment.

## 1. Introduction

Iron deficiency (ID) and heart failure (HF) often coexist and the combination of the two conditions manifests with worse outcomes. Approximately 50% of patients with HF are iron-deficient with a slightly higher prevalence in patients with heart failure with preserved ejection fraction (HFpEF) compared to their counterparts with midrange (HFmrEF) or reduced ejection fraction (HFrEF) [1,2,3]. In an international cohort of over 1500 patients with HF, ID defined as a ferritin level < 100 μg/L or ferritin 100−299 μg/L with a transferrin saturation < 20% was present in 50% of patients [4]. The prevalence was over 60% in a prospective cohort of patients with worsening HF (either hospitalized for HF or presenting with worsening HF in the outpatient setting) [5]. In that cohort, iron deficiency was more frequent among women and patients with peripheral or pulmonary congestion, kidney disease, lower hemoglobin or serum albumin levels, higher C-reactive protein levels, and P2Y12 inhibitor use. Iron deficiency affects a number of physiological processes, including exercise capacity (because of impaired cardiac and skeletal muscle function), altered mitochondrial function and cardiac energetics, and reduced cardiac performance during exercise or higher heart rates in patients with HFrEF [6].

Patient-oriented outcomes associated with ID in HF include lower oxygen consumption at peak exercise, worse 6-min walking distance, as well as overall worsening health-related quality of life (QoL) [7,8]. Increases in hospitalization for HF and all-cause mortality among iron-deficient patients with HF have also been reported [9,10], as well as higher risk of readmission after an episode of acute HF [11]. These adverse outcomes culminated in research for the treatment of ID in patients with HF. Several studies have demonstrated that intravenous (IV) iron infusions improve functional capacity and QoL, and reduce risk for HF hospitalization, but without reducing cardiovascular mortality [12]. Most recently, the IRONMAN trial, which evaluated a rapid IV iron supplementation strategy as a mean to reduce hospital resources utilization for IV iron supplementation, demonstrated a neutral impact of IV iron on cardiovascular death and an encouraging but statistically borderline effect on HF hospitalizations [13]. However, an updated meta-analysis of IV iron supplementation trials in HF, including the results of IRONMAN, demonstrated a clear effect on HF hospitalizations but not on CV or all-cause mortality [14]. Oral iron supplementation on the other hand, is not supported by the current evidence, given the lack of benefit in patients with HF, likely secondary to poor absorption of oral iron and its inability to replete the iron stores in these patients [15,16]. The poor absorption may be partially explained by higher levels of hepcidin inhibition in patients with HF, especially in patients with HFrEF [16,17].

Even though the gold standard for diagnosing ID entails bone marrow aspiration with specific staining of iron, it is limited secondary to its invasive nature and cost. Current American and European guidelines on HF define ID are defined by the following parameters: serum ferritin < 100 ng/mL or the combination of ferritin between 100−299 ng/mL and transferrin saturation (TSAT) < 20%; however, only European Society of Cardiology (ESC) guidelines recommend screening and treating HF patients for ID regardless of the presence of anemia [18,19]. These values differ significantly from the World Health Organization’s definition of ID (serum ferritin < 15 ng/mL), given the pro-inflammatory state of HF. Ferritin levels can be altered by activation of inflammatory pathways, which poses limitations when assessing ID in HF. Newer iron metabolism biomarkers may assist in redefining risk associated with ID among patients with HF, as well as identifying those that may benefit from supplementation. These biomarkers include transferrin saturation (TSAT) as a standalone marker, soluble transferrin receptor (sTfR), serum iron, and hepcidin. In this paper, we summarize the evidence linking markers of iron metabolism with outcomes in patients with HF, including all-cause mortality, HF hospitalization, functional capacity, QoL, and cardiovascular events.

## 2. Methods

### 2.1. Data Source/Search Strategy

The systematic review was conducted in accordance with the PRISMA guidelines (https://prisma-statement.org accessed on 21 November 2022). PRISMA statement and checklist are available in the Supplementary Materials (Tables S1 and S2). The search strategy identified: (1) studies in patients with HF (using the term “heart failure” in the title or abstract), with (2) suspected or established diagnosis of ID (with or without anemia), that (3) had available iron metabolism biomarkers (using appropriate terms in the title or the abstract), and (4) had available clinical outcomes, as described below. Iron metabolism biomarkers were identified by the following PubMed search terms: ferritin, hepcidin, serum iron, TSAT, and soluble transferrin receptor. PubMed was searched from inception until 17 September 2022. There was no distinction made with regard to left ventricular ejection fraction (LVEF) or other patient or HF characteristics. References of included articles were also screened for additional relevant studies. Studies were restricted to the English language. Rayyan (https://www.rayyan.ai) was used to collect, review, and select the relevant articles.

### 2.2. Study Selection

**Figure 1:**
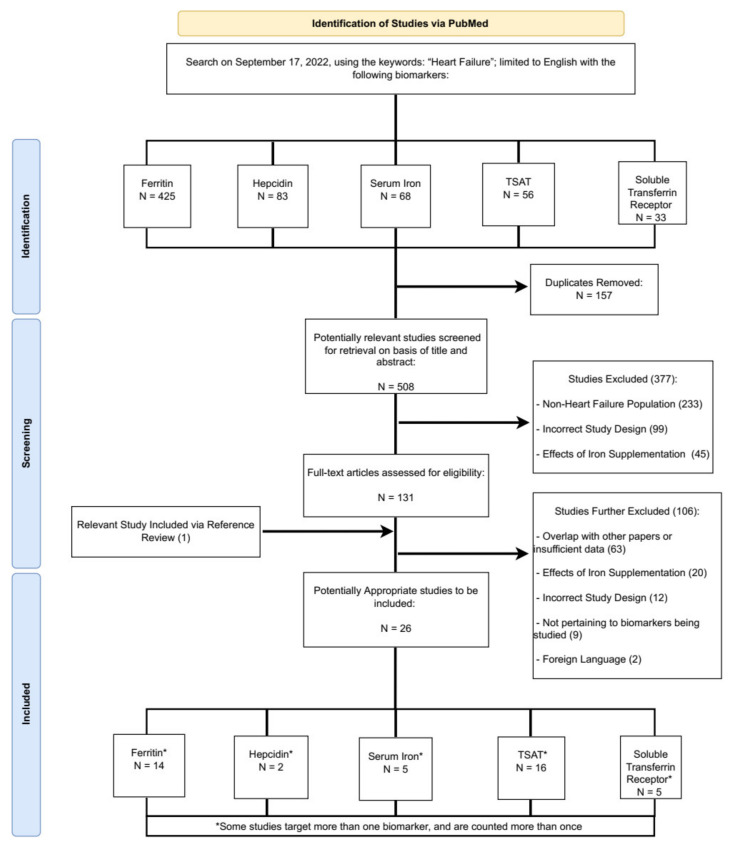
Summary of the study inclusion/exclusion process.

The workflow for study selection and the rationale for excluded studies are summarized in Figure 1. Once duplicates were removed, both investigators (AK and SD) reviewed all titles and abstracts retrieved by the literature search. Articles that did not appear to be potentially relevant to the study area, in terms of patient population, exposures of interest (iron metabolism biomarkers), and outcomes of interest, were subsequently removed. Study abstracts were independently assessed against the following inclusion criteria: (a) cohort of adult patients diagnosed with HF, (b) quantitative data on serum iron metabolism biomarkers or ranges of these biomarkers used for definition of ID or other relevant exposure, and (c) report of the outcomes of interest (mortality, hospitalization rates, functional capacity, QoL, and cardiovascular events). 

Exclusion criteria included: (a) studies not pertaining to ID or iron metabolism biomarkers, (b) biomarkers studied after pharmaceutical intervention (e.g., iron supplementation), (c) inadequate data on baseline characteristics or outcomes, (d) published solely as an abstract, and (e) non-English studies with no English translation available. We excluded interventional studies (e.g., clinical trials with iron supplementation) as our goal was to summarize the association of iron metabolism biomarkers with outcomes as a mean to examine the value of these biomarkers for patient selection (for interventions) and prognostic evaluation. As interventions likely alter the association between the iron metabolism biomarker and outcomes, we opted to exclude studies after iron supplementation to avoid interactions with the exposure of interest (biomarkers) and potential bias on the prognostic associations. Of note, these associations are not commonly reported in clinical trials, and rarely, if ever, reported per arm (so that, for example, the placebo arm can be used to evaluate the association).

### 2.3. Study Classification

Differences regarding the suitability and design of the studies were resolved by discussion between the two reviewers to reach a final classification. Studies were classified into one or more of the following outcome definitions:

- All-Cause Mortality
- HF hospitalization
- Functional Capacity/Quality of Life
- Additional Adverse Outcomes

### 2.4. Study Quality/Data Synthesis

Only studies that reported appropriate data, inclusion/exclusion criteria and baseline comparability among the studied cohorts were included in this systemic review. Moreover, the studies were screened for explicit statements regarding diagnostic criteria for heart failure, definition of iron deficiency, and length of follow-up. The heterogeneity among studies was examined by qualitative comparison of the associations of iron metabolism biomarkers with the outcomes of interest. The association of iron metabolism biomarkers with all-cause mortality and HF hospitalization was reported via relative risk estimates (hazards ratio, odds ratio, or risk rate) per biomarker unit where available, or per dichotomous definition, when no continuous estimates were provided. Functional capacity, QoL, and additional health-related and resource utilization metrics were summarized using appropriate measures for each endpoint. The above stated data was then summarized in respective tables based on outcomes of interest.

### 2.5. Risk of Bias

The data in this systematic review was conducive to formal assessment of risk of bias via the use of the Newcastle-Ottawa Scale (Supplementary Tables S3 and S4). Both investigators (AK and SD) independently reviewed the selected titles. All disagreements regarding risk of bias were resolved by discussion between the two reviewers.

## 3. Results

The initial search resulted in a total of 665 studies, with 157 duplicate studies identified, leaving 508 deduplicated articles. After full-text screening based on inclusion/exclusion criteria listed above, 26 studies remained, 4 of which were retrospective of Figure 1.

The earliest study was published in 2010 and the most recent in 2022 [20,21]. Patient inclusion criteria were reasonably similar across all studies, and most of the studies relied on the ESC guidelines for identifying ID [18]. Four studies excluded patients with anemia [4,22,23,24]. Despite the roughly similar inclusion criteria, the mean age of patients ranged from 53–79 years of age (with occasional studies reporting a median value instead of the mean). Male patients composed 41–100% of the reported population across the studies. In terms of LVEF, fifteen studies focused on reduced LVEF; two studies reported midrange LVEF, and three studies focused on preserved LVEF. Of the remaining six studies, five did not make key distinctions based on LVEF and one did not report a median/mean LVEF. These demographic and clinical characteristics are included in the corresponding tables.

### 3.1. All-Cause Mortality

The most common reported outcome was all-cause mortality in relation to iron metabolism biomarkers (*n* = 16) (Table 1). The association between ID, defined by a combination of serum ferritin and TSAT levels, with all-cause mortality in HFrEF has been demonstrated in two prospective studies [4,20]. Only one study reported the association of ferritin as a standalone marker with all-cause mortality in HFrEF, with ferritin levels <100 μg/L associated with 50% higher mortality [9]. However, low TSAT (<20%) demonstrated a stronger association with mortality in HFrEF when compared to ferritin, with a hazard ratio as high as 3.38 [25,26,27,28,29]. Low TSAT was associated with all-cause mortality in populations with midrange to preserved LVEF also [21,30,31,32]. In addition to mortality, low TSAT has been linked with the need for heart transplant and ventricular assist device implantation in patients with HFrEF [28].

Other iron metabolism biomarkers have also been evaluated for association with HF mortality. Several studies have demonstrated the predictive power of high sTfR with regard to morality in HFrEF with a cutoff value > 2.78 mg/L for 1-year mortality and >1.25 mg/L for at least 3-year mortality [33,34]. In addition, sTfR > 1.76 was strongly associated with all-cause mortality in patients with HFpEF [21].

Two studies have demonstrated the predictive value of low hepcidin for all-cause mortality; both studies focused on patients with HFrEF. The cut-off values for hepcidin differed between the studies, with reported values as high as ≤31 ng/mL and as low as <7.84 ng/mL [33,35]. The hazard ratios were 6.16 and 2.89, respectively.

Low serum iron, irrespective of ferritin and hemoglobin levels, has been associated with all-cause mortality as well. The serum cut-off values ranged from 64 µg/dL (equivalent to 11.5 μmol/L) to 13 μmol/L. No distinction was made with regard to LVEF in the studies, and hazard ratios ranged from 1.37 to 2.39 [26,32,36]. 

### 3.2. Heart Failure Hospitalization

ID, but not anemia, has been related to increased risk of hospitalizations secondary to HF exacerbation. Five studies discussed the relationship between iron metabolism biomarkers and hospitalizations secondary to HF exacerbations (Table 2). Two studies focused on ferritin, and levels < 100 μg/L were associated with increased readmission risk, with a hazard ratio approximately around 1.5 for both studies, but the length of follow-up differed substantially between the two studies, from 30 to 365 days [9,11]. Recently, two studies reported that TSAT < 20% was a stronger predictor of long-term HF hospitalizations compared to ferritin in HFrEF, with a follow-up period of 365 and 730 days [29,30]. Finally, one study demonstrated that serum iron < 11.5 μmol/L was associated with HF hospitalizations in patients with midrange LVEF, with a hazard ratio of 1.50 [36].

### 3.3. Exercise/Functional Capacity and Quality of Life

Seven studies have investigated the association between iron metabolism biomarkers and exercise/functional capacity and QoL in HF patients (Table 3). Four of the studies focused on HFrEF, while the remaining three focused on HF with midrange to preserved LVEF. Patients with ferritin < 100 μg/L had worse functional capacity measured with peak VO2 on cardiopulmonary exercise test, six-minute walk test, as well as inspiratory muscle weakness [22,25,37,38,39,40]. Lower QoL was observed with lower ferritin levels as well, independent of TSAT levels [40]. Peak oxygen consumption has been noted to be correlated with lower TSAT levels [25,38]. Increases in TfR have been reported to predict worse functional capacity and QoL (expressed as worsening NYHA functional class) [23,25].

### 3.4. Additional Adverse Outcomes

Only one study per outcome has demonstrated associations between iron metabolism biomarkers and other outcomes of interest (Table 4). Ferritin levels have demonstrated predictive potential when it comes to new-onset HF (only in female patients), as well as acute renal injury (AKI) either as a standalone biomarker or via BNP/ferritin ratio [41,42]. Variations in serum iron levels (either <7.6 μmol/L or >19.37 μmol/L) have been associated with adverse cardiovascular and cerebrovascular events in HF patients [24].

## 4. Discussion

This systematic review evaluated the role of iron metabolism biomarkers in predicting adverse outcomes in patients with HF. The main findings were as follows: (a) TSAT levels seem to have better predictive properties compared to other iron metabolism biomarkers, with regard to all-cause mortality, irrespective of LVEF, (b) ferritin < 100 ng/mL may serve as a predictor for HF hospitalizations in the short-term, (c) elevated sTfR and decreased ferritin levels are associated with both worsening functional capacity and QoL, (d) low serum iron may serve as a marker for adverse cardiovascular and cerebrovascular events in HF patients with midrange to preserved LVEF, and that (e) low ferritin may be a risk marker for AKI in acute HF.

Nearly half of HF patients suffer from ID (with or without anemia), and this comorbidity is independently associated with worse outcomes [43]. Current ESC guidelines for ID primarily focus on ferritin or TSAT combined with ferritin; however, given the complexities of iron metabolism and heart failure, limiting the evaluation of ID to one or two biomarkers for evaluation and prognostic purposes is probably not optimal. Of the 26 studies identified, 20 studies recognized ID based on ESC guidelines [18]. Recently, it has been demonstrated that ID diagnosed solely based on a TSAT <20% may result in a higher prevalence of ID compared to ESC criteria in decompensated HF patients [44]. This further emphasizes the importance of the definition of ID and when to pursue treatment. Our review summarizes the current evidence regarding the role of iron metabolism biomarkers in predicting outcomes in HF, which may assist in individualizing patient care.

### 4.1. Iron Metabolism and Mortality

The evidence for the role of ID in the risk of all-cause mortality has been limited. Several studies have reported that various iron metabolism biomarkers are individually associated with mortality; however, TSAT < 20% has demonstrated the greatest consistent risk [25,26,27,28,29,30,31]. Other iron metabolism biomarkers have been linked with all-cause mortality in HF as well. Of note, the latter studies are neither consistent in relating these biomarkers to all-cause mortality, nor are their effects (associated relative risks) as profound as those reported with TSAT levels [9,26,33,34,35]. The numerical cutoffs utilized to assess the effects of other iron metabolism biomarkers (especially hepcidin, sTfR, and serum iron) have not been uniform in the various studies, and therefore it is difficult to draw conclusions about an appropriate target value [21,26,33,34,35,36]. Overall, it may be beneficial to serially evaluate TSAT levels and treat to ensure levels are greater than 20%, irrespective of ferritin, to provide mortality benefits. From a therapeutic perspective, a recent meta-analysis, involving 10 studies and a total of 3373 participants, reported that IV iron supplementation in patients with HFrEF has a strong signal for a favorable effect on cardiovascular mortality with IV iron (odds ratio 0.86; 95% CI 0.70 to 1.05; *p* = 0.14) and a weaker signal for all-cause mortality (odds ratio 0.93; 95% CI 0.78 to 1.12; *p* = 0.47), but the current evidence was inconclusive and larger trails with longer follow-up would be needed [14]. Of note, in the same meta-analysis, patients with transferrin saturation <20% benefited more from iron therapy vs. those with higher saturation, but the interaction for this difference did not reach statistical significance and needs to be interpreted with caution [14].

### 4.2. Iron Metabolism and Heart Failure Hospitalizations

Regardless of anemia status, ID has been independently associated with recurrent admissions for HF [45]. This systematic review demonstrates the benefits of serially evaluating ferritin levels in the short-term and treating if less than 100 ng/mL, for it may predict HF readmissions within 30 days to 1 year [9,11]. However, when compared to ESC guidelines, including ferritin <100 ng/mL, only TSAT and serum iron levels, have demonstrated an association with increased HF hospitalization risks over a span of 2 to 3 years [29,36]. These studies demonstrate that there is no obvious iron metabolism biomarker to assess for HF hospitalization risk, but various biomarkers in combination may provide complementary information over time.

This is important, as recent data suggest a beneficial effect of IV iron supplementation on HF hospitalizations in patients with HF. A recent meta-analysis suggests that iron therapy, in the form of repeat IV doses, leads to a substantial ~25% reduction in HF hospitalizations in patients with HFrEF [14]. This meta-analysis included a total of 3373 participants from 10 trials, and approximately 50% were assigned to IV iron therapy. There was strong evidence that IV iron therapy reduced the composite endpoint of recurrent HF hospitalization or cardiovascular mortality (rate ratio 0.77; 95% CI 0.66 to 0.90; *p* = 0.001) and first hospitalization for HF or cardiovascular death (odds ratio 0.74; 95% CI 0.63−0.87; *p* < 0.001). These favorable effects were driven by the 27% reduction in HF hospitalizations observed in the 3 largest studies (rate ratio 0.73; 95% CI 0.62 to 0.86; *p* < 0.001) [13,46,47]. The effect was similar both for first and recurrent events, indicating a durable benefit. This magnitude of event reduction matches that of other “pillar” therapies in HFrEF (angiotensin-renin system inhibitors, beta blockers, aldosterone antagonists, and sodium glucose cotransporter 2 inhibitors). Notably, in the two largest trials to date, IRONMAN and AFFIRM-AHF, this favorable effect was achieved with only a few IV iron therapy sessions. In AFFIRM-AHF, there were two repletion doses plus hemoglobin-guided boosters [47], and in IRONMAN, 78% of patients received ≤2 infusions over a median of 2.7 years) [13], which has major implications for the practicality of adopting IV iron repletion more broadly.

### 4.3. Iron Metabolism and Other Outcomes

A negative correlation between ID and exercise endurance has further been established [48]. A “depleted stores” status of various iron metabolism biomarkers has consistently demonstrated worse functional capacity [38]. ID may lead to cardiac damage and dysfunction, a byproduct of energy disruptions and impairment in oxidative metabolism [43]. As a result of the ESC guideline recommendations for ID assessment, ferritin levels have been studied in more detail. Once again, worse HF symptoms and exercise intolerance have been reported with low ferritin in patients with HF, assessed with six-minute walk tests and cardiopulmonary exercise tests, the latter demonstrating reduced peak VO2 also with low ferritin [22,37,38,39]. In addition to worse functional capacity, decreased ferritin stores have been linked with poorer QoL, a pattern that has been seen with elevated sTfR levels also [23,25,40]. These results emphasize the ability of a less commonly evaluated iron metabolism biomarker to communicate multiple facets of a patient’s well-being. Of note, IV iron therapy, in addition to a substantial favorable effect on HF hospitalizations (and potentially, cardiovascular mortality), improves exercise capacity and quality of life, independently of anemia correction [14].

In addition to functional capacity, an interdependent relationship between the heart and kidney exists, also known as the cardiorenal syndrome (CRS) [49]. ID may lead to an exacerbation of HF and worsening CRS via a vicious cycle related to tissue hypoxia causing release of nitric oxide, which leads to peripheral dilation and hypotension. In turn, the sympathetic nervous system is activated, renal vasoconstriction takes place with both the reduction of renal function and activation of the renin-angiotensin aldosterone system [50]. Traditionally, natriuretic peptides like B-type natriuretic peptide (BNP) are used to assess acute HF exacerbations, and at times, serially evaluated to guide decongestion strategy. However, the relationship between ID and CRS suggests that iron metabolism biomarkers may assist in guiding treatment of acute HF, and prevent adverse outcomes, such as AKI. Ferritin, either as a standalone marker, or evaluated as the ratio between BNP and ferritin, may assist in the detection of patients at risk for developing AKI in the setting of acute HF [42]. That study demonstrated that ferritin may assist in directing diuresis during HF exacerbation. However, as this use of ferritin has not been reported by other groups, further research is needed before recommending use in clinical practice.

Moreover, HF patients with ID are at increased risk of cardiovascular events [20,25]. Of the iron metabolism biomarkers, only serum iron has been studied in association with prognostication of such adverse events taking place [24]. A specific cutoff value has not been recognized, as increased risks of cardiovascular events were seen with both depleted and elevated iron levels. However, although serum iron levels are often ordered with the traditional ferritin and TSAT to assess for ID, many times the values are overlooked. The results from this review once again emphasize approaching ID from a holistic perspective and emphasize the role of less “traditional” iron metabolism biomarkers in assessing risk for adverse outcomes of HF.

### 4.4. Future Directions

ID is a common and detrimental comorbidity in HF. Hence, treatment of ID is an important goal in HF therapy and may serve as a protective factor for various adverse outcomes. Especially relevant are different diagnostic criteria for ID in HF patients in order to ensure at-risk patients are appropriately treated.

Despite the encouraging results of a recent meta-analysis on the effect of IV iron therapy for iron-deficient patients with HF [14], several questions regarding this form of therapy remain unanswered. For example, the evidence for patients with HFpEF is limited to subgroup analyses from clinical trials that did not specify LVEF-based entry criteria, and the total number of patients with HFpEF in those studies was small. Similarly, LVEF criteria were uncommon in the observational studies that we included in this review. Therefore, the findings of this review, which connects iron metabolism markers with outcomes, and the recent meta-analysis on the effects of IV iron in HF, apply mostly to patients with at least mildly reduced LVEF. We need more evidence before we can extend these findings (and recommendations) to patients with HFpEF. The ongoing IRONMET-HFpEF trial (NCT04945707) will provide insights into the effect of IV iron on exercise capacity and quality of life in patients with HFpEF. Cardiovascular mortality benefits with IV iron therapy still require a more definitive clinical study with longer-term follow-up, as most trials to date had ≤12 months of follow up. The ongoing HEART-FID trial (NCT03037931), which has enrolled >3000 iron-deficient patients, will evaluate the effects of 6-monthly IV iron infusions on a hierarchical primary composite endpoint of death and HF hospitalization at 12 months and change from baseline to 6 months in the 6-min walk test distance. The ongoing FAIR-HF2 trial (NCT03036462) will evaluate the effect of 4-monthly FCM infusions on the combined rate of recurrent HF hospitalizations and cardiovascular death in patients with HFrEF, with a minimum average follow-up of 2 years.

## 5. Limitations

Our review has a number of limitations. First, the included studies were both retrospective and prospective; however, some studies did not report multivariate adjusted rate ratios or hazard ratios or length of the study. Second, the studies were heterogeneous with regard to biomarkers studied. Third, the study populations primarily consisted of male patients and therefore the results may not be generalizable to female patients. Fourth, very few studies differentiated based on LVEF; thus, it was difficult to establish and assess if the relationship between the biomarker’s predictive value was altered based on LVEF. Fifth, very few studies specified the subtype of cardiovascular event that led to mortality (e.g., myocardial infarction, stroke, pulmonary embolism, etc.). Sixth, only a few acute HF studies specified whether hospitalization for HF was de novo or decompensation of existing HF. Finally, there was not a uniform method in testing functional capacity among the various studies.

## 6. Conclusions

Our present systematic review has important clinical implications. A substantial number of patients with HF have ID, and the biomarkers utilized for assessing the extent of ID may have implications in predicting adverse outcomes. TSAT levels < 20% seem to be superior in predicting all-cause mortality and long-term HF hospitalizations. Ferritin and sTfR predict both functional capacity and QoL. In addition, ferritin may also assess the likelihood of developing AKI in the setting of acute decompensated HF. These findings warrant further evaluation of a wider use of iron metabolism biomarkers for clinical decision making in patients with HF. As evidence continues to mount in support of the strategy of maintaining normal iron levels with IV iron repletion as a way of reducing adverse events in HFrEF, it would be important for prospective studies to include the entire spectrum of iron metabolism biomarkers and report mechanistic endpoints (echocardiographic and exercise capacity metrics) to facilitate optimization of patient selection.

## Figures and Tables

**Table 1:** Characteristics of Studies Associating Biomarkers of Iron Metabolism with All-Cause Mortality.

Author (Year)	N	LVEF (%)	Mean Age (Years)	Male (%)	Follow-Up Period (Days)	Study Type	ID Criteria	Anemia Excluded (Yes/No)	Biomarker(s)	Outcomes	Association of Iron Metabolism Markers with Outcomes
Jankowska (2010) [20]	546	26 ± 7	55 ± 11	88	731 ± 350 (mean)	P	ESC definitions	No	Ferritin TSAT	Mortality: 28% in 2 years	↑ morality or HT w/ID, based on ferritin and TSAT (HR 1.58, 95% CI 1.14–2.17, *p* < 0.01)
Okonko (2011) [25]	157	32 ± 9	71 ± 12	72	743 (median)	P	TSAT <20%	No	TSAT	Mortality: 17% in 2 y	↑ mortality w/TSAT <20% (HR 3.38; 95% CI 1.48−7.72; *p* = 0.004)
Jankowska (2013) [35]	321	31 ± 9	61 ± 11	84	1095 (endpoint)	P	N/A	No	Hepcidin	Mortality: 24% in 3 years	↑ mortality w/Serum hepcidin ≤31 ng/mL (HR 2.89, 95%CI 1.63–5.05, *p* < 0.001)
Klip (2013) [4]	1506	33 ± 14	64 ± 13	74	701 (median)	P	ESC definitions	Yes (Hg < 8−10 g/dL based on cohort)	Ferritin TSAT	Mortality: 29.2% in 2 years	↑ morality w/ID, based on ferritin and TSAT (HR 1.42, 95%CI 1.14−1.77, *p* = 0.002)
Jankowska (2014) [33]	165	33 ± 13	65 ± 12	81	323 ± 94 (mean)	P	(a) Hepcidin <14.5 ng/mL; (b) sTfR ≥1.59 mg/L; (c) ESC definitions	No	Hepcidin sTfR	Mortality in low hepcidin AND high sTfR: 41% in 1 year	↑ morality w/Low Hepcidin <7.84 ng/mL and High sTfR >2.78 mg/L (HR = 6.16, 95% CI = 2.70–14.04, *p* < 0.001)
Grote Beverborg (2018) [26]	387	30.4 ± 9.3	66.8 ± 13.4	68	746 (endpoint)	P	ESC definitions	No	Iron TSAT	Mortality: 25% in 1.5 years	↑ mortality w/serum iron ≤13 µmol/L (HR 2.39; 95% CI 1.13–5.04; *p* = 0.022); ↑ mortality w/TSAT <20% (HR 2.78; 95% CI 1.22–6.34, *p* = 0.015); Ferritin <100 ng/mL not significantly associated w/mortality (HR 1.54; 95% CI 0.45–5.52)
Nakano (2018) [9]	578	39 (median)	78 (median)	61	365 (endpoint)	P	ESC definitions	No	Ferritin	Mortality: 11% in 1 year	↑ adverse events w/Ferritin <100 μg/L (HR 1.50, 95% CI 1.02–2.21, *p* = 0.040); FID with neutral effect (HR 0.73, 95% CI 0.39–1.34, *p* = 0.31)
Ambrosy (2020) [30]	4103	35% with LVEF > 50%	79.2 ± 7	51	365 (endpoint)	P	ESC definitions	No	TSAT	Mortality and HF Hospitalization: 16.7 per 100 person-years	↑ mortality w/TSAT < 20% compared to ferritin <100 ng/mL (aHR 1.42, 95% CI:1.20–1.68) and ferritin 100–300 ng/mL (aHR 1.18, 95% CI:1.00–1.38)
Kurz (2020) [28]	2223	34 ± 15	57.2 ± 14.7	72	2068 (median)	P	ESC definitions	No	TSAT	Mortality: 8% in 5.6 years	↑ mortality, HT, VAD implant w/TSAT < 20% (HR 0.608, 95% CI 0.498–0.741, *p* < 0.001) Neutral Effect when comparing ferritin levels 100–300 μg/L vs. <100 μg/L (HR 0.857, 95% CI 0.643–1.144, *p* = 0.298)
Gentil (2020) [27]	108	28.1 ± 9.2	59.5 ± 14.1	52.8	857.5 (median)	P	ESC definitions	No	TSAT	Mortality: 29% in 2.3 years	↑ mortality w/TSAT < 20% (HR = 2.3; 95% CI: 1.11–4.85, *p* = 0.026); Ferritin < 100 μg/L was not related to mortality (Log-rank test, *p* = 0.439)
Sierpinski (2020) [34]	791	28 ± 8	58 ± 11	84	1095 (endpoint)	P	based on ROC for ferritin, TSAT, sTfR	No	sTfR	Mortality: 34% in 3 years	↓ Survival Rate w/sTfR ≥ 1.25 mg/L: (Z = 4.03, *p* < 0.001)
Fitzsimons (2021) [29]	1563	38 *	65.6 *	72	730 (endpoint)	P	ESC definitions	No	TSAT Ferritin	Mortality: 19% in 2 years	↑ mortality w/TSAT < 20% (HR 1.95, CI 95% 1.52−2.51, *p* < 0.01): ESC definitions had inferior effect (HR 1.21, CI 95% 0.95–1.53, *p* = 0.12)
Palau (2021) [31]	1701	51.7% with LVEF > 50%	76 (median)	56	30 (endpoint)	P	ESC definitions	No	TSAT	Mortality: 6% in 1 month	↑ adverse outcomes w/TSAT, exponentially higher if TSAT < 10% (HR 1.75, CI 95% 1.08–2.83; *p* = 0.024); Ferritin levels, even <54 ug/L, without higher risk (HR 0.97, CI 95% 0.63–1.50; *p* = 0.906)
Ueda (2021) [36]	615	46.7 ± 16.0	74.3 ± 12.0	57	976 (mean)	P	ESC definitions	No	Iron	Mortality: 25% in 2.7 years	↑ adverse outcomes w/serum iron ≤ 64 µg/dL (irrespective of ferritin/Hg) (HR 1.500; CI 95% 1.128–1.976; *p* = 0.0044)
Fitzsimons (2022) [21]	788	36% with LVEF ≥ 50%	70.0 *	69	1460 (endpoint)	P	(1) sTfR ≥ 1.59 mg/L, (2) sTfR ≥ 1.76 mg/L (3) ESC definitions (4) TSAT < 20%	No	sTfR TSAT	Mortality and HF hospitalizations: 54% in 4 years	↑ mortality w/sTfR > 1.76 mg/L in HFpEF (HR 1.84, 95% CI 1.23–2.75); ↑ mortality w/sTfR > 1.59 mg/L regardless LVEF (HR 1.45, 95% CI 1.13–1.86); ↑ mortality w/TSAT < 20% in HFpEF (HR, 1.69, 95% CI 1.10–2.59); ESC definitions were not associated with mortality (HR 1.19, 95% CI 0.77–1.85)
Masini (2022) [32]	4422	41% with LVEF ≥ 50%	75 (median)	60	1490 (median)	P	most accurate biomarker of iron status (ferritin, TSAT, iron)	No	Iron TSAT	Mortality: 34.5% in 5 years	↑ mortality w/TSAT < 20%(HR: 1.27; 95% CI: 1.14−1.43; *p* < 0.001); ↑ mortality w/Serum Iron ≤13 μmol/(HR: 1.37; 95% CI: 1.22−1.54; *p* < 0.001); Serum ferritin < 100 ng/mL insignificantly associated with lower mortality (HR: 0.91; 95% CI: 0.81−1.01; *p* = 0.09)

↑: increase; ↓: Decrease; Study Type: (P) prospective, (R) retrospective; (Mean) ± (Standard Deviation), unless otherwise specified; * Average calculation among the various study groups; Adverse events: combination of death and HF hospitalization; CI: confidence interval; ESC Definitions: ferritin < 100 ng/mL or ferritin 100–300 ng/mL AND TSAT < 20%; FID (functional iron deficiency): ferritin 100–300 ng/mL AND TSAT < 20%; HR: hazard ratio; HF: heart failure; HT: heart transplant; Hg: hemoglobin; ID: iron deficiency; LVEF: left ventricular ejection fraction; ROC: receiver operating characteristic curve; sTfR: soluble transferrin receptor; TSAT: transferrin saturation; VAD: ventricular assisted device.

**Table 2:** Characteristics of Studies Associating Biomarkers of Iron Metabolism with Heart Failure Hospitalization.

Author (Year)	N	LVEF (%)	Mean Age (Years)	Male (%)	Follow-Up Period (Days)	Study Type	ID Criteria	Anemia Excluded (Yes/No)	Biomarker(s)	Outcomes	Association of Iron Metabolism Markers with Outcomes
Núñez (2016) [11]	626	52.1% with LVEF >50%	73.4 ± 10.4	52	30 (endpoint)	P	ESC definitions	No	Ferritin	HF Hospitalization: 16.5% in 30 days	↑ HF Hospitalization w/ferritin < 100 µg/L (sHR 1.72; 95% CI 1.13–2.60, *p* = 0.011); FID not related to the risk of readmission (HR 0.87; 95% CI 0.46–1.62, *p* = 0.652)
Nakano (2018) [9]	578	39 (median)	78 (median)	61	365 (endpoint)	P	ESC definitions	No	Ferritin	HF Hospitalization: 19% in 1 year	↑ Adverse events w/Ferritin < 100 μg/L (HR 1.50, 95% CI 1.02–2.21, *p* = 0.040) FID with neutral effect (HR 0.73, 95% CI 0.39–1.34, *p* = 0.31)
Ambrosy (2020) [30]	4103	35% with LVEF >50%	79.2 ± 7	51	365 (endpoint)	P	ESC definitions	No	TSAT	HF admission plus mortality: 16.7 per 100 person-years	↑ HF Hospitalization w/TSAT < 20% for ferritin <100 ng/mL (aHR 1.40, 95% CI 1.16–1.70) and 100–300 ng/mL (aHR 1.24, 95% CI 1.01–1.52)
Fitzsimons (2021) [29]	1563	38 *	65.6 *	72	730 (endpoint)	P	ESC definitions OR TSAT< 20%	No	TSAT	Death/HF hospitalization: 43% in 2 years	↑ Adverse events w/TSAT < 20% (HR 1.19, CI 1.01–1.41, *p* = 0.03) ESC definitions not a predictor of hospitalization (HR 1.11, CI 95% 0.92–1.33, *p* = 0.28)
Ueda (2021) [36]	615	46.7 ± 16.0	74.3 ± 12.0	57	976 (mean)	P	ESC definitions	No	Iron	HF hospitalization: 29% in 2.7 years	↑ Adverse events w/Serum iron ≤ 64 µg/dL, irrespective of ferritin/Hg (HR 1.500; 95% CI 1.128–1.976; *p* = 0.0044)

↑: increase; Study Type: (P) prospective, (R) retrospective; (Mean) ± (Standard Deviation), unless otherwise specified; * Average calculation among the various study groups; Adverse events: combination of death and HF hospitalization; CI: confidence interval; ESC Definitions: ferritin < 100 ng/mL or ferritin 100–300 ng/mL AND TSAT < 20%; FID (functional iron deficiency): ferritin 100–300 ng/mL AND TSAT < 20%; HR: hazard ratio; HF: heart failure; Hg: hemoglobin; ID: iron deficiency; LVEF: left ventricular ejection fraction; TSAT: transferrin saturation.

**Table 3:** Characteristics of Studies Associating Biomarkers of Iron Metabolism with Exercise/Functional Capacity and Quality of Life.

Author (Year)	N	LVEF (%)	Mean Age (Years)	Male (%)	Follow-Up Period (Days)	Study Type	ID Criteria	Anemia Excluded (Yes/No)	Biomarker(s)	Outcomes	Association of Iron Metabolism Markers with Outcomes
Jankowska (2011) [37]	443	26 ± 7	54 ± 10	90	--	P	ESC definitions	No	Ferritin	Functional Capacity	↓ VO2 and higher VE/VCO2 slope with serum ferritin (*p* < 0.05); ESC definitions associated with reduced peak VO_2_ (β = −0.14, *p* < 0.01 *p* < 0.05) and higher VE-VCO_2_ slope (β = 0.14, *p* < 0.01 *p* < 0.05)
Okonko (2011) [25]	157	32 ± 9	71 ± 12	72	743 (median)	P	TSAT < 20%	No	sTfR TSAT Ferritin	Functional Capacity Quality of Life	↑ NYHA class with sTfR ≥1.76 mg/L (ANOVA *p* < 0.0001) VO_2_ positively correlated with TSAT (r = 0.71, *p* < 0.0001) and ferritin (r = 0.48, *p* = 0.01) levels
Pozzo (2017) [22]	138	28.1 *	61.2 *	48	1825 (mean)	P	ESC definitions	Yes (Hg < 13.0 g/dL in men or <12.0 g/dL in women)	Ferritin	Functional Capacity	↓ performance in 6MWT (*p* = 0.03) and ↓ VO_2_ (*p* = 0.01) with ferritin < 100 μg/L
Martens (2018) [38]	1197	6% with LVEF > 50%	70 ± 12	71	1004 (mean)	P	ESC definitions	No	Ferritin TSAT	Functional Capacity	↓ VO_2_ with ID, based on ferritin and TSAT (*p* < 0.001)
Tkaczyszyn (2018) [39]	53	LVEF ≤ 40	64 ± 10	100	--	P	ESC definitions	No	Ferritin	Functional Capacity	↓ MIP with ferritin <100 μg/L compared to ferritin ≥100 μg/L (r = 0.42, *p* < 0.01)
Bekfani (2019) [40]	190	58 ± 7	71 ± 9	67.5	--	P	ESC definitions	No	Ferritin TSAT	Functional Capacity Quality of Life	↓ performance in 6MWT (*p* = 0.008) and CPX (*p* = 0.03) with ID, based on ferritin and TSAT ↓ QoL(*p* = 0.03) with Ferritin < 100 µg/L
Alcaide-Aldeano (2020) [23]	447	62 ± 8	76 ± 9	41	--	P	ferritin < 100 ng/mL or TSAT < 20%	Yes (Hg less than < 8.5 g/dL)	sTfR	Functional Capacity Quality of Life	sTfR (overall mean value of 1.92) predictor of functional capacity (β = −63, *p* < 0.0001, R2 0.39); and QoL (β = 7.95, *p* < 0.0001, R2 0.14). TSAT < 20% (β = 3.32, *p* = 0.13, R2 0.10), ferritin (β = 0.64, *p* = 0.56, R2 0.10) and serum iron (β = −1.50, *p* = 0.47, R2 0.10) did not have a strong association to QoL

↑: increase; ↓: Decrease; Study Type: (P) prospective, (R) retrospective; (Mean) ± (Standard Deviation), unless otherwise specified; * Average calculation among the various study groups; CPX: Cardiopulmonary exercise test; CI: confidence interval; ESC Definitions: ferritin < 100 ng/mL or ferritin 100–300 ng/mL AND TSAT < 20%; HR: hazard ratio; HF: heart failure; ID: iron deficiency; LVEF: left ventricular ejection fraction; NYHA class: New York Heart Association class; QoL: quality of life; MIP: maximal inspiratory pressure at the mouth; ROC: receiver operating characteristic curve; 6MWT: six-minute walk test; sTfR: soluble transferrin receptor; TSAT: transferrin saturation; VE/VCO2: carbon dioxide production; VO2: peak oxygen consumption.

**Table 4:** Characteristics of Studies Associating Biomarkers of Iron Metabolism with Additional Adverse Outcomes.

Author (Year)	N	LVEF (%)	Mean Age (Years)	Male (%)	Follow-Up Period (Days)	Study Type	ID Criteria	Anemia Excluded (Yes/No)	Biomarker(s)	Outcomes	Association of Iron Metabolism Markers with Outcomes
Klip (2017) [41]	6386	N/A	53.1 ± 12.0	50.3	3032 (median)	P	N/A	No	Ferritin	New onset HF	ferritin levels (median: 97 µg/L) predict new-onset HF in women only (*p* = 0.024).
Yan (2020) [24]	221	55.8 ± 9.1	68.6 ± 11.8	57.5	239 (median)	R	Low (7.6 ± 1.6), medium (11.9 ± 1.8) and high (19.4 ± 3.8 serum iron (μmol/L)	Yes	Iron	MACCE	↑ MACCE with serum iron <7.58 μmol/L or >19.37 μmol/L compared to medium serum iron (*p* < 0.0001)
Ceyhun (2021) [42]	157	30.0 ± 8.1	63.9 ± 8.62	42	N/A	R	N/A	No	Ferritin	AKI	↑ AKI risk with BNP/ferritin, mean value 10.48 ± 2.14 (OR = 3.19; 95% CI 1.92–6.54; *p* = 0.001) & ferritin, mean value 86.78 ± 57.2 (OR = 0.72; 95% CI, 0.89–0.53; *p* = 0.028)

↑: increase; Study Type: (P) prospective, (R) retrospective; (Mean) ± (Standard Deviation), unless otherwise specified; AKI: acute kidney injury BNP: B-type natriuretic peptide; CI: confidence interval; HR: hazard ratio; HF: heart failure; ID: iron deficiency; LVEF: left ventricular ejection fraction; MACCE: major adverse cardiovascular and cerebrovascular events.

## Data Availability

Data are available per request on https://www.rayyan.ai.

## Supplementary Materials

The following supporting information can be downloaded at: https://www.mdpi.com/article/10.3390/ijms24065645/s1.

## Abbreviations

AKI	Acute renal injury
BNP	Brain natriuretic peptide
CRS	Cardiorenal syndrome
ESC	European Society of Cardiology
HF	Heart failure
HFmrEF	Heart failure with midrange ejection fraction
HFpEF	Heart failure with preserved ejection fraction
HFrEF	Heart failure with reduced ejection fraction
ID	Iron deficiency
IV	Intravenous
LVEF	Left ventricular ejection fraction
QoL	Quality of life
STfR	Soluble transferrin receptor
TSAT	Transferrin saturation
