# Supplementary material for: Markers of Iron Metabolism and Outcomes in Patients with Heart Failure: A Systematic Review
Source: Int J Mol Sci. 2023 Mar 15;24(6):5645. doi: 10.3390/ijms24065645 (PMC10059277; doi:10.3390/ijms24065645)
Supplement: Supplementary file 1 [file ijms-24-05645-s001.zip › ijms-2206827-SI.pdf]

Part I: PRISMA Statement

Supplementary Table S1: PRISMA 2020 Abstract Checklist, comprised of 12 items to ensure a comprehensive, relevant, and coherent abstract summarizing the systemic review.

Section and Topic	Item #	Checklist item	Location where item is reported
**TITLE**			
Title	1	Identify the report as a systematic review.	Page 1
**BACKGROUND**			
Objectives	2	Provide an explicit statement of the main objective(s) or question(s) the review addresses.	Page 1; lines 12-14
**METHODS**			
Eligibility criteria	3	Specify the inclusion and exclusion criteria for the review.	Page 1; lines 17-21
Information sources	4	Specify the information sources (e.g. databases, registers) used to identify studies and the date when each was last searched.	Page 1; lines 14-17
Risk of bias	5	Specify the methods used to assess risk of bias in the included studies.	Page 1; lines 21-22
Synthesis of results	6	Specify the methods used to present and synthesize results.	Page 1; lines 22-23
**RESULTS**			
Included studies	7	Give the total number of included studies and participants and summarise relevant characteristics of studies.	Page 1; lines 22-25
Synthesis of results	8	Present results for main outcomes, preferably indicating the number of included studies and participants for each. If meta-analysis was done, report the summary estimate and confidence intervals. If comparing groups, indicate the direction of the effect (i.e. which group is favoured).	Page 1; lines 25-36
**DISCUSSION**			
Limitations of evidence	9	Provide a brief summary of the limitations of the evidence included in the review (e.g. study risk of bias, inconsistency and imprecision).	N/A; Page 12

Section and Topic	Item #	Checklist item	Location where item is reported
Interpretation	10	Provide a general interpretation of the results and important implications.	Page 1; lines 37-42
**OTHER**			
Funding	11	Specify the primary source of funding for the review.	N/A; Page 12
Registration	12	Provide the register name and registration number.	N/A

Supplementary Table S2: PRISMA 2020 Checklist, comprised of of 27 items utilized to improve transparency and optimize the quality of reporting within systemic reviews.

Section and Topic	Item #	Checklist item	Location where item is reported
**TITLE**			
Title	1	Identify the report as a systematic review.	Page 1
**ABSTRACT**			
Abstract	2	See the PRISMA 2020 for Abstracts checklist.	Page 1; S1 Table
**INTRODUCTION**			
Rationale	3	Describe the rationale for the review in the context of existing knowledge.	Page 2
Objectives	4	Provide an explicit statement of the objective(s) or question(s) the review addresses.	Page 2
**METHODS**			
Eligibility criteria	5	Specify the inclusion and exclusion criteria for the review and how studies were grouped for the syntheses.	Page 3

Section and Topic	Item #	Checklist item	Location where item is reported
Information sources	6	Specify all databases, registers, websites, organisations, reference lists and other sources searched or consulted to identify studies. Specify the date when each source was last searched or consulted.	Page 3
Search strategy	7	Present the full search strategies for all databases, registers and websites, including any filters and limits used.	Page 3
Selection process	8	Specify the methods used to decide whether a study met the inclusion criteria of the review, including how many reviewers screened each record and each report retrieved, whether they worked independently, and if applicable, details of automation tools used in the process.	Page 3
Data collection process	9	Specify the methods used to collect data from reports, including how many reviewers collected data from each report, whether they worked independently, any processes for obtaining or confirming data from study investigators, and if applicable, details of automation tools used in the process.	Page 3
Data items	10a	List and define all outcomes for which data were sought. Specify whether all results that were compatible with each outcome domain in each study were sought (e.g. for all measures, time points, analyses), and if not, the methods used to decide which results to collect.	Page 4-5
	10b	List and define all other variables for which data were sought (e.g. participant and intervention characteristics, funding sources). Describe any assumptions made about any missing or unclear information.	Page 5
Study risk of bias assessment	11	Specify the methods used to assess risk of bias in the included studies, including details of the tool(s) used, how many reviewers assessed each study and whether they worked independently, and if applicable, details of automation tools used in the process.	Page 5, S2 Figure, S3 Table
Effect measures	12	Specify for each outcome the effect measure(s) (e.g. risk ratio, mean difference) used in the synthesis or presentation of results.	Page 6
Synthesis methods	13a	Describe the processes used to decide which studies were eligible for each synthesis (e.g. tabulating the study intervention characteristics and comparing against the planned groups for each synthesis (item #5)).	Page 3-5
	13b	Describe any methods required to prepare the data for presentation or synthesis, such as handling of missing summary statistics, or data conversions.	Page 5
	13c	Describe any methods used to tabulate or visually display results of individual studies and syntheses.	Page 5
	13d	Describe any methods used to synthesize results and provide a rationale for the choice(s). If meta-analysis was performed, describe the model(s), method(s) to identify the presence and extent of statistical heterogeneity, and software package(s) used.	Page 5

Section and Topic	Item #	Checklist item	Location where item is reported
	13e	Describe any methods used to explore possible causes of heterogeneity among study results (e.g. subgroup analysis, meta-regression).	Page 5
	13f	Describe any sensitivity analyses conducted to assess robustness of the synthesized results.	N/A
Reporting bias assessment	14	Describe any methods used to assess risk of bias due to missing results in a synthesis (arising from reporting biases).	Page 5, S2 Figure, S3 Table
Certainty assessment	15	Describe any methods used to assess certainty (or confidence) in the body of evidence for an outcome.	Page 5
**RESULTS**			
Study selection	16a	Describe the results of the search and selection process, from the number of records identified in the search to the number of studies included in the review, ideally using a flow diagram.	Page 6, Figure 1
	16b	Cite studies that might appear to meet the inclusion criteria, but which were excluded, and explain why they were excluded.	Figure 1
Study characteristics	17	Cite each included study and present its characteristics.	Table 1-4
Risk of bias in studies	18	Present assessments of risk of bias for each included study.	S2 Figure, S3 Table
Results of individual studies	19	For all outcomes, present, for each study: (a) summary statistics for each group (where appropriate) and (b) an effect estimate and its precision (e.g. confidence/credible interval), ideally using structured tables or plots.	Table 1-4
Results of syntheses	20a	For each synthesis, briefly summarize the characteristics and risk of bias among contributing studies.	S2 Figure, S3 Table
	20b	Present results of all statistical syntheses conducted. If meta-analysis was done, present for each the summary estimate and its precision (e.g. confidence/credible interval) and measures of statistical heterogeneity. If comparing groups, describe the direction of the effect.	Page 5-7
	20c	Present results of all investigations of possible causes of heterogeneity among study results.	N/A
	20d	Present results of all sensitivity analyses conducted to assess the robustness of the synthesized results.	N/A
Reporting biases	21	Present assessments of risk of bias due to missing results (arising from reporting biases) for each synthesis assessed.	N/A

Section and Topic	Item #	Checklist item	Location where item is reported
Certainty of evidence	22	Present assessments of certainty (or confidence) in the body of evidence for each outcome assessed.	N/A
**DISCUSSION**			
Discussion	23a	Provide a general interpretation of the results in the context of other evidence.	Page 7-9
	23b	Discuss any limitations of the evidence included in the review.	Page 10
	23c	Discuss any limitations of the review processes used.	Page 10
	23d	Discuss implications of the results for practice, policy, and future research.	Page 9, 10
**OTHER INFORMATION**			
Registration and protocol	24a	Provide registration information for the review, including register name and registration number, or state that the review was not registered.	N/A
	24b	Indicate where the review protocol can be accessed, or state that a protocol was not prepared.	Page 10
	24c	Describe and explain any amendments to information provided at registration or in the protocol.	N/A
Support	25	Describe sources of financial or non-financial support for the review, and the role of the funders or sponsors in the review.	Page 10
Competing interests	26	Declare any competing interests of review authors.	Page 10
Availability of data, code and other materials	27	Report which of the following are publicly available and where they can be found: template data collection forms; data extracted from included studies; data used for all analyses; analytic code; any other materials used in the review.	S1 Figure; Table 1-4

Part II: PubMed Search Strategy

Supplemental Methods: Search strategy employed for this systemic review.

Run on September 17, 2022:

heart failure [tiab] AND ferritin [tiab] AND eng [la]
heart failure [tiab] AND hepcidin [tiab] AND eng [la]
heart failure [tiab] AND iron [tiab] AND eng [la]
heart failure [tiab] AND transferrin [tiab] AND eng [la] *
heart failure [tiab] AND TSAT [tiab] AND eng [la]

(*captures: transferrin, transferrin saturation, and soluble transferrin receptor)

Part III: Assessment of Potential Bias

Supplementary Table S3: The Newcastle-Ottawa Scale quality instrument, modified for this systemic review. Each study is scored by awarding a point for each answer that is marked with an asterisk below. Possible total points are: 4 points for Selection, 2 points for Comparability, and 3 points for Outcomes.

SELECTION:

1. **Representativeness of the Exposed Cohort**
    a. Truly representative of the average patient with heart failure (*e.g*, with regard to severity of illness, comorbidities) in the community*
    b. Somewhat representative of the average patient with heart failure (*e.g.* with regard to severity of illness, comorbidities) in the community*
    c. Selected group of users (e.g. volunteers, pregnant, elderly, significant physical disabilities)
    d. No description of the derivation of the cohort
2. **Selection of the Non-Exposed Cohort**
    a. Drawn from the same community as the exposed cohort*
    b. Drawn from a different source*
    c. No description of the derivation of the non-exposed cohort
3. **Ascertainment of Exposure**
    a. Secure record (e.g. medical records)*
    b. Structured interview*
    c. Written self-report
    d. No description
4. **Demonstration that Outcome of Interest Was Not Present at Start of Study**
    a. Yes*
    b. No

COMPARABILITY

1. **Comparability of Cohorts on the Basis of the Design or Analysis**

    a. study controls for age/sex (the most important factor)*

    b. Study controls for any additional factor* (this criteria can be modified to indicate a specific control for a second important factor)

    c. Inadequate degree of control

OUTCOME

1. **Assessment of Outcome**

    a. Independent or blind assessment stated in the paper, or confirmation of the outcome by reference to secure records (x-rays, medical records, etc)*

    b. Record linkage (e.g, identified through ICD codes on database records)*

    c. Self-report (e.g no reference to original medical records or x-rays to confirm the outcome)

    d. No description

2. **Was Follow-up Long Enough for Outcomes to Occur?**

    a. Yes (select an adequate follow up period for outcome of interest)*

    b. No

3. **Adequacy of Follow-up of Cohorts**

    a. Complete follow-up—all subjects accounted for*

    b. Subjects lost to follow-up unlikely to introduce bias—small number lost (LESS than 20% follow-up, or description provided of those lost)*

    c. Follow-up rate MORE than 20% and no description of those lost

    d. No statement

    If <20% of subjects were lost to follow-up, but the difference between groups was large, study was downgraded to ‘c,’ especially if no reasons for difference in follow-up are provided.

Supplementary Table S4: Risk of Bias score for each of the 26 studies based on the Newcastle-Ottawa Scale quality instrument.

**Study**	**Year**	**SELECTION**				**COMPARABILITY**	**OUTCOME**			**Total Quality Score**
		Representative of the Exposed Cohort	Non-exposed cohort from same community as exposed	Secure record (e.g. medical records) or structured interview	Demonstration that Outcome of Interest Was Not Present at Start of Study	Comparability of Cohorts based on the Design or Analysis	Assessment of Outcome	Was Follow-up Long Enough for Outcomes to Occur?	Adequacy of Follow-up of Cohorts	
Jankowska [21]	2010	*	*	*	*	**	*	*	*	9
Jankowska[39]	2011	*	*	*	*	**	*			8
Okonko[27]	2011	*	*	*	*	**	*	*	*	9
Jankowska[37]	2013	*	*	*	*	**	*	*	*	9
Klip[23]	2013	*	*	*	*	**	*	*	*	9
Jankowska[35]	2014	*	*	*	*	**	*	*	*	9
Núñez[11]	2016	*	*	*	*	**	*	*	*	9
Klip[43]	2017	*	*	*	*	**	*	*	*	9
Pozzo[24]	2017	*	*	*	*	**	*	*	*	9
Grote Beverborg[28]	2018	*	*	*	*	**	*	*		8
Martens[40]	2018	*	*	*	*	**	*	*		8
Nakano[9]	2018	*	*	*	*	**	*	*	*	9
Tkaczyszyn[41]	2018		*	*	*	*	*			5
Bekfani[42]	2019	*	*	*	*	**	*			7
Alcaide-Aldeano[25]	2020	*	*	*	*	**	*			7
Ambrosy[32]	2020	*	*	*	*	**	*	*		8
Gentil[29]	2020	*	*	*	*	**	*	*	*	9
Kurz[30]	2020	*	*	*	*	**	*	*	*	9
Sierpinski[36]	2020	*	*	*	*	**	*	*		8
Yan[26]	2020	*	*	*	*	**	*	*	*	9
Ceyhun[44]	2021	*	*	*	*	**	*			7
Fitzsimons[31]	2021	*	*	*	*	**	*	*		8
Palau[33]	2021	*	*	*	*	**	*	*		8
Ueda[38]	2021	*	*	*	*	**	*	*		8
Fitzsimons[22]	2022	*	*	*	*	**	*	*		8
Masini[34]	2022	*	*	*	*	**	*	*		8
